# *Tbx16* and *mesogenin 1* promote presomitic mesoderm differentiation by repressing the mesodermal progenitor cell state

**DOI:** 10.1371/journal.pgen.1012176

**Published:** 2026-06-08

**Authors:** Guoyu Zhu, Miriam A. Genuth, Yanrong Xiao, Abigail A. Kindberg, Kayleigh Hackett, Scott A. Holley

**Affiliations:** Department of Molecular, Cellular and Developmental Biology, Yale University, New Haven, Connecticut, United States of America; Geisel School of Medicine at Dartmouth, UNITED STATES OF AMERICA

## Abstract

During zebrafish embryonic body elongation, differentiation of mesodermal progenitors into presomitic mesoderm requires the transcription factors *tbx16* and *mesogenin 1*. Here, by using temporally controlled *tbx16* and *mesogenin 1* overexpression and RNAseq to identify immediate downstream changes in gene expression, we elucidate how these genes promote presomitic mesoderm differentiation. Using machine learning and game theory, we integrated differentially expressed genes with wild-type scRNAseq data and identified genes downstream of *tbx16* and *mesogenin 1* during mesoderm differentiation. This data-driven analysis indicates that *mesogenin 1* and *tbx16* primarily repress expression of genes as mesodermal progenitors differentiate. Strikingly, the genes that are most important for defining transcriptional cell states during mesoderm differentiation are most strongly repressed by *tbx16* and *mesogenin 1*. Moreover, these downstream effectors are enriched for genes with known roles in mesoderm development and body elongation such as Fgf, Wnt and Bmp pathways and the transcription factors *tbxta*, *eve1*, *hoxd12a*, *hoxd13b*, *lef1*, *cdx4*, *tbx16l*, *ved*, *vent* and *vox*. Gradients of Fgf and Wnt specify the mesodermal progenitor state in the posterior tailbud and activate many of these transcription factors indicating that *tbx16* and *mesogenin 1* promote mesoderm differentiation by repressing this progenitor state.

## Author summary

Mesodermal progenitor cells continuously disperse and differentiate during vertebrate body elongation and give rise to the muscle, bone and dermis of the trunk and tail. In zebrafish, two genes, the transcription factors *tbx16* and *mesogenin 1*, function semi-redundantly during differentiation of mesodermal progenitors. However, the genes downstream of *tbx16* and *mesogenin 1* that mediate mesoderm morphogenesis remain unknown. To identify these downstream genes, we performed RNA sequencing shortly after over-expression of *tbx16* or *mesogenin 1*. These types of experiments typically provide a long list of genes, and it is challenging to identify the most important of the downstream genes for further analysis. We address this challenge by integrating the RNA sequencing data with single cell RNA sequencing data of wild-type mesoderm differentiation. We employ machine learning to sort gene expression into distinct states and game theory to identify the most important genes for defining each state. Comparing these genes with the gene expression changes after *tbx16* or *mesogenin 1* over-expression, we identify a smaller number of genes that are enriched in genes with known functions in mesodermal differentiation. This suggests that this data analysis strategy can identify important biological information from large genomic datasets.

## Introduction

As the post-gastrulation vertebrate embryo elongates, it forms the posterior trunk and tail concomitant with segmentation of the paraxial mesoderm into somites. The tailbud is the posterior growth zone and contains progenitors of the spinal cord, vertebral column and skeletal muscle. Genetic analyses and live imaging experiments of the zebrafish tailbud found that posterior body elongation is largely driven by cell migration and not cell proliferation [1–5]. Neuromesodermal progenitors (NMP) in the dorsal medial posterior tailbud differentiate into either spinal cord or mesodermal progenitors [6–8]. NMPs that differentiate into mesodermal progenitors undergo an epithelial to mesenchymal transition (EMT) then migrate ventrally into the progenitor zone, where they exhibit disordered cell motion [8–11]. These cells subsequently assimilate into the presomitic mesoderm (PSM) and cell movement diminishes as the tissue solidifies [1,12,13]. This developmental progression has been demarcated by machine learning into a series of cell state transitions in both gene expression and cell motion as NMPs differentiate first into mesodermal progenitors, then into a motile progenitor zone, then into posterior PSM and then anterior PSM [14]. Here, we examine the roles of two transcription factors, *tbx16* and *mesogenin 1*, in paraxial mesoderm differentiation.

Previous studies indicate that *tbx16* and *msgn1* have both independent and redundant functions in paraxial mesoderm development. Loss of either *tbx16* or *msgn1* function leads to a failure of PSM differentiation with loss of *tbx16* having a much stronger phenotype [15–19]. *Tbx16* was first identified as a recessive lethal mutation in zebrafish called *spadetail (spt-1*) [15]. Embryos homozygous for this mutation exhibit a bent posterior body, an accumulation of cells in the posterior tailbud incapable of transitioning into PSM, and a corresponding lack of somitic mesoderm. Subsequently, the gene mutated in *spadetail* was identified as a t-box transcription factor, named *tbx16*, with homologs in *Xenopus* and chick [16,20–24]. In the zebrafish embryo, *tbx16* is expressed in the mesodermal progenitors, the PZ domain and the posterior PSM [16]. *Tbx16* genetically interacts with other t-box transcription factors *tbxta* (*brachyury/no tail*) and *tbx6* within a gene network to regulate mesoderm development [16,17,25,26]. Wnt and FGF signaling activate expression of *tbxta* and *tbx16* to promote NMP differentiation into mesodermal progenitors [8,9,16,27,28]. *Tbx16* regulates *hox* gene expression in the tailbud and induces intermediate mesodermal fates by regulating Wnt, retinoic acid and FGF signaling [29–31]. As a transcription factor, Tbx16 can act as both an activator and a repressor. In the zebrafish gastrula, Tbx16 directly activates *myf5* and *myoD* transcription downstream of FGF during myogenesis [32]. In the tailbud, Tbx16 represses *sox2* transcription to drive NMPs to differentiate as mesoderm [28]. Despite these extensive analyses, we still do not know the genes downstream of *tbx16* that carryout differentiation.

*Mesogenin* is a basic-helix-loop-helix (bHLH) transcription factor expressed in the tailbud and first identified in chick [33]. In mouse and *Xenopus*, *mesogenin* is crucial for paraxial mesoderm specification [34,35]. Zebrafish *mesogenin 1* (*msgn1*) is expressed in the posterior tailbud, in the mesodermal progenitors, PZ domain and posterior PSM in a pattern very similar to that of *tbx16*. *Msgn1* expression is sharply reduced in *spt* mutant embryos suggesting that *msgn1* is downstream of *tbx16* [36]. Further studies in *Xenopus* and mouse showed that *msgn1*’s expression in the PSM is controlled by *tbx6* and Wnt signaling [37–41].

Embryos lacking both *tbx16* and *msgn1* function display a similar, but more severe phenotype to embryos lacking *tbx16* or *msgn1*, with a mass of mesodermal progenitors accumulating in the posterior tailbud and lack of somitic mesoderm [18,19]. These studies suggest that *tbx16* and *msgn1* repress the mesodermal progenitor state, but it remains unclear how they regulate mesoderm morphogenesis. *Tbx16;msgn1* double mutants have significantly more mesodermal progenitor cells, posterior spinal cord progenitor cells, and fewer muscle cells compared to wild type [42]. Mesodermal progenitors lacking *tbx16* and *msgn1* fail to produce functional lamellipodia to establish directional migration despite being highly mobile [43]. In mice, *tbx6*;*msgn1* and double mutants exhibit a similar genetic redundancy in regulating mesoderm development in the tailbud [44]. In zebrafish, *tbx6l* can partially compensate for loss of *tbx16* function [45].

It is still unclear how *tbx16* and *msgn1* regulate the morphogenesis and differentiation of the paraxial mesoderm. Indeed, it is challenging for gene expression analysis to explain phenotypes given the large number of genes and their non-linear interactions and the extensive variability observed both across expression states and among individual cells. This complexity is further compounded by the uneven functional importance of genes with similar expression patterns. Here, we propose a data-driven algorithm that applies the Shapley value [46], a well-established game theory metric, to the analysis of gene expression data. This algorithm not only identifies key genes involved in cell state transitions but also quantitatively estimates the correlation between overall gene expression levels across cells and the progression of cell differentiation. This work provides a systematic method to elucidate the relationship between gene expression patterns and developmental transitions. Moreover, the generalizability of this data-driven framework allows for its adaptation to other types of gene expression data and diverse biological processes.

In this study, we employ machine learning and game theory to analyze the gene network that governs zebrafish body elongation and paraxial mesoderm development. We perform temporally controlled overexpression of *tbx16* and *msgn1* during zebrafish body elongation and examine downstream gene expression using RNA sequencing on pooled dissected tailbuds. We analyzed the bulk RNA sequencing results by combining them with scRNAseq of the wild-type cell state transitions. This analysis reveals that *tbx16* and *msgn1* promote mesodermal differentiation primarily by inhibition of the mesodermal progenitor gene regulatory network. While *tbx16* and *msgn1* regulate many of the same genes, *tbx16* uniquely regulates more genes than *msgn1*. This study contributes new insights not only into vertebrate body elongation and mesodermal development but also into the methodology of gene expression data analysis.

## Results

### Transgene overexpression of *tbx16* or *msgn1*

**Fig 1:**
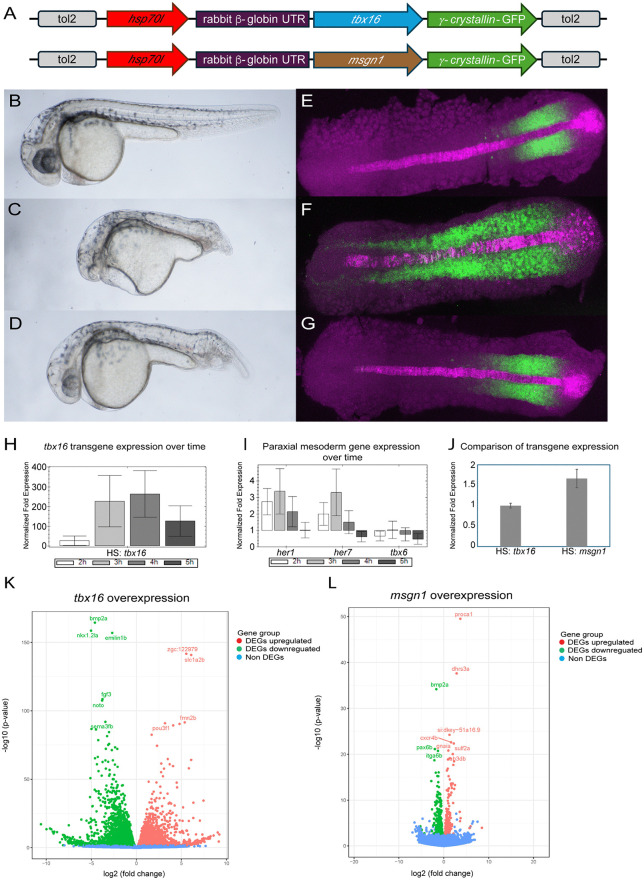
Heat-shock over-expression of *tbx16* or *msgn1* alters gene expression within 3 hours. **(A)** *hsp70-*driven *tbx16* and *msgn1* transgenic constructs. *Tol2* sites at both ends of the construct allow for *tol2* transposase-mediated insertion into the genome. The gamma-crystallin promoter drives GFP expression in the lens to be used for screening. The rabbit beta globin sequence is used as a unique sequence to measure transgene mRNA. B-D. Embryos 1 day after 2 ~ 3 somite stage heat shock. **(B)** a wild-type embryo. **(C)** a tg(*hsp70l-tbx16*) embryo. **(D)** a tg(*hsp70l-msgn1*) embryo. **(E-G)** Fluorescent in situ hybridization of *tbx6* (green) and *tbxta* (magenta) in wild-type **(E)** tg(*hsp70l:tbx16*), and tg(*hsp70l:msgn1*) embryos. **(H)** RT-qPCR analysis for *beta globin* sequence of the 5’UTR of transgenic constructs at different times after heat shock. **(I)** RT-qPCR analysis *her1, her7* and *tbx6* from wild-type, *tg(hsp70l:tbx16)*, and *tg(hsp70l:msgn1)* embryos. *Beta-actin* is used as a reference for to normalize for total amount of mRNA in each isolate. Analysis was performed on *hsp70l*: *tbx16* embryos 2, 3, 4, 5 hours post heat shock. Fold change is relative to expression levels of WT embryos at two hours post heat shock. **(J)** Tg(*hsp70l-msgn1*) exhibits higher expression than tg(*hsp70l-tbx16*) 3 hours after heat shock. **(K-L)** Volcano plot of RNA-seq experiments to embryo dissected tailbuds of *tbx16* overexpression vs wild type **(K)** and *msgn1* overexpression vs wild type **(L)**. The upregulated, downregulated, and non DEGs are color coded as red, green and blue dots, respectively.

We generated transgenic zebrafish lines using the *tol2* transposase system to overexpress either *tbx16* or *msgn1* under the *hsp70l* heat-shock promoter (Fig 1A) [47,48]. We heat-shocked transgenic embryos at the 2–3 somite stage at 38º C for 30 minutes. There are significant phenotypic differences between heat shocked transgenic and wild-type embryos 24 hours after heat shock (Fig 1C and 1D). Compared to wild-type embryos (Fig 1B), tg(*hsp70l:tbx16*) embryos have a severely truncated tail, which is expected since *tbx16* represses *tbxta* expression [9,16,18,19,28,49]. *tbx16* overexpression also disrupts eye and head development (Fig 1C). Overall, tg(*hsp70l:msgn1*) embryos have a less severe phenotype. In these embryos, anterior morphology is similar to that of wild-type embryos, but the posterior body is truncated and lacks a notochord (Fig 1D).

We next sought to define the timing of gene expression changes that underlie these morphological phenotypes. First, we determined the earliest timepoint after heat shock at which significant alterations in gene expression can be detected. We performed RT-qPCR experiments on tg(*hsp70l:tbx16*) embryos 2, 3, 4, 5 hours after heat shock. We used the rabbit *beta globin* sequence in our transgene (Fig 1A) to measure heat shock induction of transgene mRNA expression. *her1*, *her7*, *tbx6* mRNA were assayed as markers of paraxial mesoderm differentiation and *beta-actin* was used as a control [50]. We used intron qPCR primers for *her1* and *her7* to measure nascent transcription and exon primers for *tbx6* and *beta-actin*. tg(*hsp70l:tbx16*) transgene expression is strongly induced 3 hours after heat shock and peaks 4 hours after heat shock (Fig 1H). *her1* and *her7* transcription increases most at 3 hours after heat shock, but *tbx6* expression did not exhibit a significant change in mRNA levels compared to wild type (Fig 1I). We compared the relative expression of the transgenes 3 hours after heat shock and found that the tg(*hsp70l:msgn1*) displayed higher fold induction relative to the tg(*hsp70l:tbx16*) (Fig 1J). Based on these results, we performed subsequent experiments 3 hours after heat shock to identify the initial burst of gene expression changes following transgene induction.

We examined spatial changes in gene expression using fluorescent in situ hybridization of *tbx6* and *tbxta* on wild-type, tg(*hsp70l:tbx16*) and tg(*hsp70l:msgn1*) embryos 3 hours after heat shock (Fig 1E-1G). Compared to wild-type embryos, tg(*hsp70l:tbx16*) embryos extend the expression of *tbx6* anteriorly, and *tbxta* expression is more variegated in the posterior tailbud. In tg(*hsp70l:msgn1*) embryos, *tbx6* expression is slightly extended to the anterior, and *tbxta* expression in the posterior tailbud is attenuated compared to wild type. This effect of *msgn1* over-expression is weaker than the phenotype produced by a prior tg(*hsp70l:msgn1*) transgene which induced a larger anterior expansion of *tbx6* expression [18]. Overall, the tg(*hsp70l:tbx16*) transgene has a greater effect on both body elongation and tailbud gene expression than tg(*hsp70l:msgn1*).

### RNAseq of transgenic tailbuds

Our aim was to reveal the downstream effectors of *tbx16* and *msgn1* that mediate mesodermal progenitor differentiation. The next step in this process was to identify genes that rapidly respond to over-expression of *tbx16* and *msgn1*. We used bulk RNA sequencing to maximize read depth. We performed RNA sequencing of pooled dissected tailbuds of wild type, tg(*hsp70l:tbx16*) and tg(*hsp70l:msgn1*) embryos 3 hours after heat shock. We performed three biological replicates of each genotype. To identify significant differentially expressed genes, we used DESeq2 with an adjusted p-value cutoff of 0.05 and a 1.5-fold change in expression. We identified 6976 differentially expressed genes (DEGs) in tg(*hsp70l:tbx16*) embryos and 706 in tg(*hsp70l:msgn1*) embryos compared to wild type (Fig 1K-1L and S1 Fig and S1 and S2 Tables). The larger number of DEGs in tg(*hsp70l:tbx16*) embryos is consistent with its more broad and severe phenotype compared to tg(*hsp70l:msgn1*) embryos. As expected, *tbxta* is downregulated by 3.31 fold in tg(*hsp70l:tbx16*) embryos which corresponds to the severely truncated tail phenotype. In tg(*hsp70l:msgn1*) embryos, *noto* expression is downregulated by 1.73 fold which accounts for the lack of posterior notochord [51].

### Gene expression analysis via DESeq2

**Fig 2:**
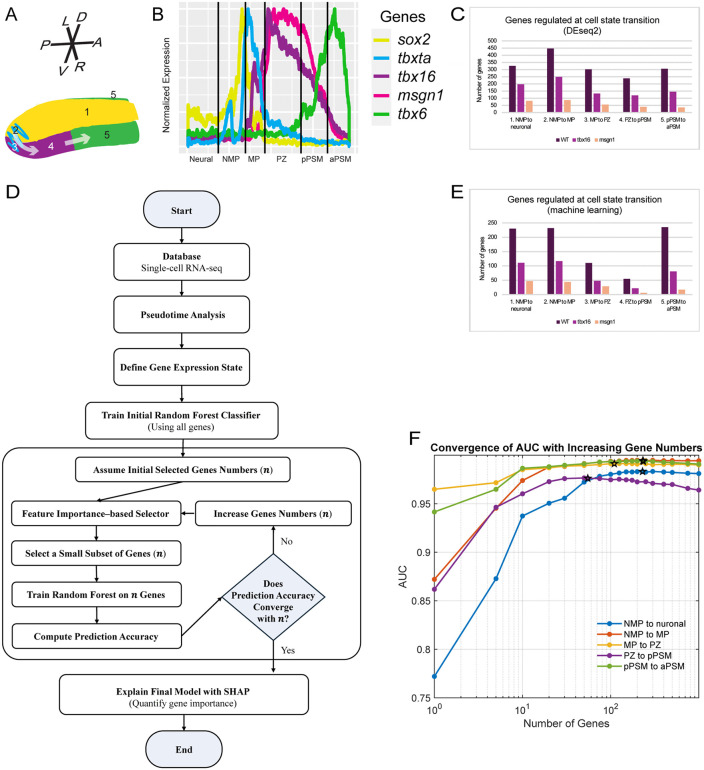
Overview of machine-learning-based selection of cell state transition genes. **(A)** A schematic showing the developmental trajectory of the paraxial mesoderm in the tailbud. Body axes are shown D (dorsal), V (ventral), A (anterior), P (posterior), R (right) and L (left). The expression patterns of *sox2* (yellow), *brachyury* (cyan), *tbx16* (purple), and *tbx6* (green) are schematized. **(B)** Smoothed, normalized single cell gene expression profiles of wild type tailbuds cells over the pseudotime trajectory, where optimal change points delineate six gene expression states: neuronal, neuromesodermal progenitors (NMP), mesodermal progenitors (MP), progenitors zone (PZ), posterior (pPSM) and anterior PSM (aPSM). NMPs can develop either as neuronal cells (leftward) or as mesodermal progenitors (rightward). Five representative genes from 10269 identified genes are shown. **(C, E)** Histogram showing the number of genes exhibiting expression changes during each wild-type cell state transition. Also shown is the overlap with genes regulated by *tbx16* or *msgn1* overexpression compared to wild type (≥1.5-fold change). **(C)** Genes identified by DESeq2 using a 1.5-fold expression change threshold during the cell state transitions. **(D)** The flowchart of the data driven framework to identify key genes correlated to the gene expression state transitions and to quantify each gene’s importance with a Shapley value. **(E)** Genes identified by the machine learning algorithm. **(F)** Random forest model training accuracy on the test dataset versus the number of genes selected (x-axis in log scale). The star marker indicates the number of genes selected by the random forest classifier that maximizes prediction accuracy under optimal hyperparameters.

We proceeded to analyze the correlation between the genes misregulated by *tbx16* or *msgn1* overexpression with the genes that change expression levels during paraxial mesoderm development. To that end, we utilized the scRNAseq expression data and paraxial mesoderm cell state classification from [14]. In that study, UMAP was used to arrange the cells in a one-dimensional pseudotime sequence, and then the pseudotime was segmented into distinct transcriptional cell states using a variance minimization algorithm. That protocol identified six different cell states: neuronal, neuromesodermal progenitor (NMP), mesodermal progenitor (MP), progenitor zone (PZ), posterior (pPSM) and anterior PSM (aPSM), (Fig 2A and 2B). To identify genes differentially expressed during this developmental progression, we pooled gene expression in individual cells by cell state and experimental replicate, i.e., pseudobulking, and performed DESeq2 comparisons between each adjacent pair of pseudotime segments. The number of differentially expressed genes ranged from 243 at the PZ to pPSM transition to 467 at the NMP to MP transition (Fig 2C and S3 Table). This gene set includes known neuronal, NMP and mesodermal genes such as *sox2, sox3, tbxta, tbx16, msgn1, tbx6* and *mespaa*. *Tbx16* and *msgn1* overexpression alter expression of different subsets of genes that change at each transition.

We generated scatterplots of these data for each cell state transition (S2 and S3 Figs and S4 and S5 Tables). These plots show how expression of each gene changes (up or down) in the scRNAseq pseudotime along the x-axis. The log-fold change in gene expression after heat shock of tg(*hsp70l:tbx16*) or tg(*hsp70l:msgn1*) is plotted along the y-axis. The preponderance of gene expression is repressed by *tbx16* overexpression (S2 Fig). We reasoned that since *tbx16* is required for paraxial mesoderm differentiation, true *tbx16* target genes would either be upregulated at a particular cell state transition in wild type (WT^+^) and also upregulated after *tbx16* overexpression (tbx16^+^) or be downregulated at a particular wild-type cell state transition (WT^-^) and also downregulated by *tbx16* overexpression (tbx16^-^). These genes are in the top right and bottom left quadrants of each scatterplot, respectively. These plots show a bias in WT^-^tbx16^-^ genes, particularly at the NMP to MP, PZ to pPSM and pPSM to aPSM transitions (S2 Fig). The *msgn1* analysis also shows enrichment of WT^-^msgn1^-^ genes particulary at the PZ to pPSM transition whereas the MP to PZ transition is enriched for both WT^-^msgn1^-^ and WT^+^msgn1^+^ genes (S3 Fig).

### Gene expression analysis using machine learning

The pseudotime analysis incorporates 21301 genes to calculate the pseudotime coordinates with breakpoints for gene expression state classification [14]. However, the high dimensionality of the data and the structure of this classification complicate quantification of how DESeq2 pseudobulked gene expression changes contribute to transitions between consecutive cell states (e.g., NMP to MP). To uncover the intricate relation between developmental gene expression state and individual genes, we utilized a Random Forest Classifier with Shapley Additive exPlanations (SHAP) framework customized for decision-tree models (Fig 2D) [52,53]. The Random Forest Classifier was used to reconstruct the gene expression states and provide probabilities missing from the orginal pseudotime states. SHAP used these probabilites to quantify the importance of each gene in defining each transcriptional state. The underlying hypothesis is that genes that are important for defining transcriptional state transitions during differentiation will be functionally important. If this hypothesis is correct, then these gene lists should be enriched for genes with known functions in paraxial mesoderm development.

The first three steps were the creation of the pseudotime-classified dataset (Fig 2D) [14]. The Random Forest Classifier was then trained on this dataset to identify a minimal subset of genes capable of accurately classifying gene expression states from the full gene set (Fig 2F). We identified a selected number of genes for each cell state with the subset size of these genes overlapping with those up- or downregulated (≥1.5-fold) by *tbx16* or *msgn1* overexpression (Fig 2D). The Random Forest Classifier gene lists are more selective than the DESeq2-generated gene lists (compare Fig 2C to 2E).

**Fig 3:**
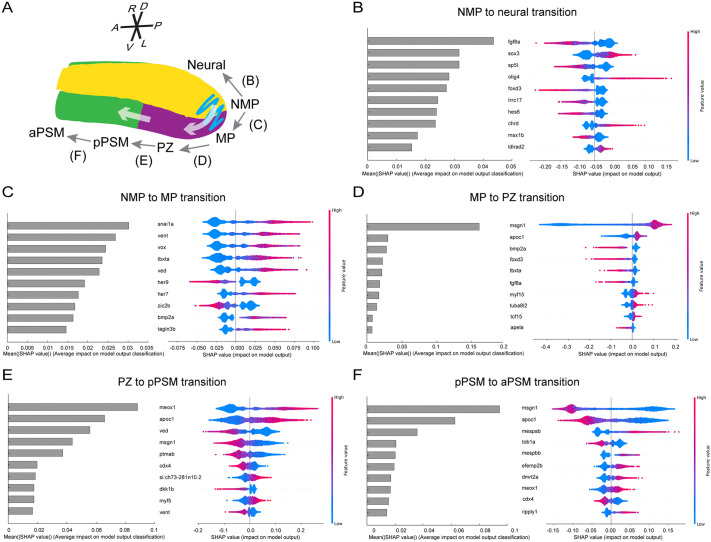
SHAP identifies the most important genes for each cell state transition. **(A)** A schematic showing the developmental trajectory of the paraxial mesoderm in the tailbud. Body axes are shown as D (dorsal), V (ventral), A (anterior), P (posterior), R (right) and L (left). Cell state transitions are shown as NMP to neural, NMP to MP, MP to PZ, PZ to pPSM, and pPSM to aPSM. Each transition is labeled with an arrow indicating the direction of transition. **(B-F)** The 10 most essential genes correlating to wild-type cell state transitions from NMP to neural **(B)**, NMP to MP **(C)**, MP to PZ **(D)**, PZ to pPSM **(E)**, and pPSM to aPSM **(F)**. These genes are ranked by their overall impact as denoted by their mean absolute Shapley value (left). The summary plot (right) combines gene importance with their overall effect on the transitions: the color represents the relative expression value of each gene from low (blue) to high (red), while 𝐱 axis (Shapley value) denotes the increase or decrease in probable impact on the cell state transition.

The resulting model structure was then used to compute gene-specific Shapley values across individual cells [46]. Shapley values are a game theory-based metric of the contribution of each component of a group to the outcome/performance of the group. The approach has recently been extensively used to interpret machine-learning model predictions to account for intricate interactions between features [53]. Here, the Shapley value represents the expected marginal contribution of a gene’s expression level to the predicted probability that a cell belongs to a particular transcriptional state, averaged over all possible combinations of other genes in the database. For each single cell, a positive Shapley value indicates that high or low expression of the gene raises the probability of the cell belonging to a particular cell state, while a negative value indicates that high or low expression of the gene reduces the probablity of the cell belonging to the state, given the influence of all other genes in the random forest decision tree model. For example, high expression of *fgf8a* decreases the probablity of a cell being neural while low expression increases the probability of a cell being neural (Fig 3B).

Application of SHAP analysis to all 8120 wild-type cells classified in the MP and PZ states with the 235 identified key genes yielded an 8120 × 235 matrix of Shapley values, representing the contributions of each gene across those wild-type cells to the decision-tree classification. Fig 3 presents summary plots that demonstrate the effect of each gene, ordered according to their importance (only the 10 most essential genes are shown). As expected, the transition between MP to PZ gene expression states is dependent upon *msgn1* in a positive relation (Fig 3D).

**Fig 4:**
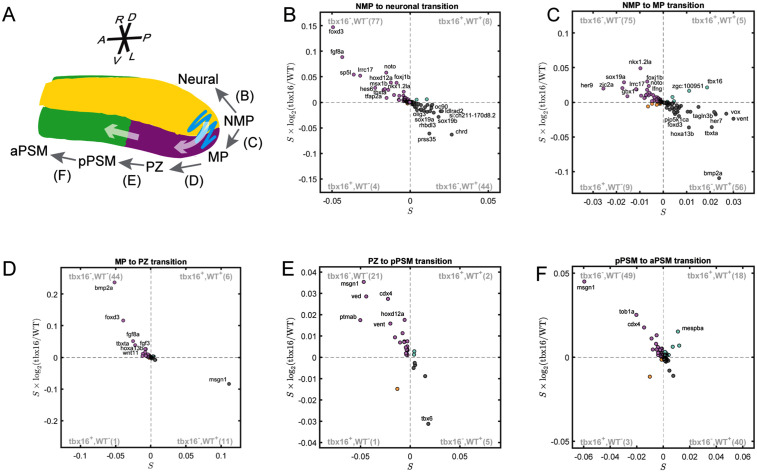
Transcriptional regulation by *tbx16* during cell state transitions of paraxial mesoderm differentiation. **(A)** A schematic showing the developmental trajectory of the paraxial mesoderm in the tailbud. Body axes are shown as D (dorsal), V (ventral), A (anterior), P (posterior), R (right) and L (left). Cell state transitions are shown as NMP to neural, NMP to MP, MP to PZ, PZ to pPSM, and pPSM to aPSM. Each transition is labeled with an arrow indicating the direction of transition. **(B-F)** The change in probability in decision tree model-predicted state transition under *tbx16* overexpression is estimated by a linear function ($𝐒\times {\text{log}}_{\text{2}}\left(\text{tbx16}/\text{WT}\right)$) of each gene’s log-scale sensitivity parameter (𝐒). Key genes identified by machine learning with positive or negative correlations to wild-type gene expression states transitions (${WT}^{+},{\text{WT}}^{-}$) and overlapping with genes up- or downregulated (≥1.5-fold; $tbx{\text{16}}^{+},\text{tbx}{\text{16}}^{+}$) under *tbx16* overexpression compared to wild type are analyzed during NMP to neural **(B)**, NMP to MP **(C)**, MP to PZ **(D)**, PZ to pPSM **(E)**, and pPSM to aPSM transitions **(F)**.

**Fig 5:**
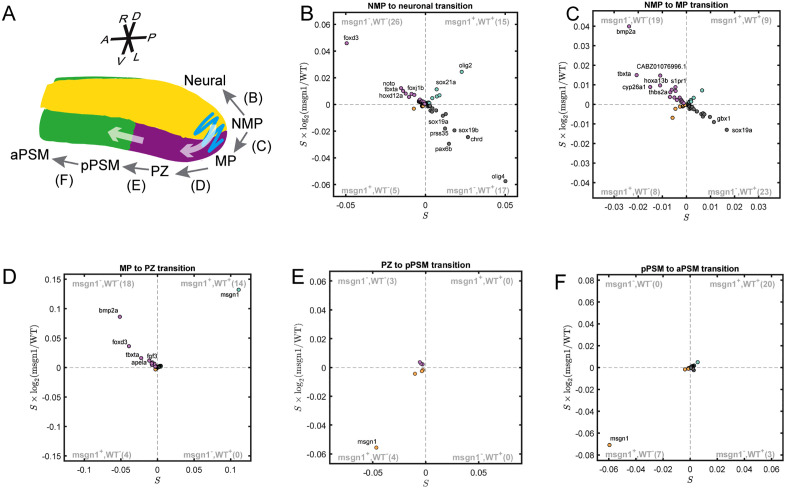
Transcriptional regulation by *msgn1* during cell state transitions of paraxial mesoderm differentiation. **(A)** A schematic showing the developmental trajectory of the paraxial mesoderm in the tailbud. Body axes are shown as D (dorsal), V (ventral), A (anterior), P (posterior), R (right) and L (left). Cell state transitions are shown as NMP to neural, NMP to MP, MP to PZ, PZ to pPSM, and pPSM to aPSM. Each transition is labeled with an arrow indicating the direction of transition. **(B-F)** The change in probability in decision tree model-predicted state transitions under *msgn1* overexpression, estimated by a linear function ($𝐒\times {\text{log}}_{\text{2}}\left(\text{msgn1}/\text{WT}\right)$) of each gene’s log-scale sensitivity parameter (𝐒). Key genes identified by machine learning with positive or negative correlations to wild-type gene expression states transitions (${WT}^{+},{\text{WT}}^{-}$) and overlapping with genes up- or downregulated (≥1.5-fold; ${msgn1}^{+},{\text{msgn1}}^{-}$) under *msgn1* overexpression compared to wild type are analyzed during NMP to neural **(B)**, NMP to MP **(C)**, MP to PZ **(D)**, PZ to pPSM **(E)**, and pPSM to aPSM transition **(F)**.

Lastly, we utilized the Shapley matrix of single cell gene expression to estimate how the bulk RNAseq gene expression after *tbx16* or *msgn1* overexpression influences the transitions of gene expression states during paraxial mesoderm differentiation. Here, we introduced a log-scale sensitivity parameter (*S*), representing the proportional change in a gene’s Shapley value per log₂-fold change in normalized expression (S6 Table). The calculation of *S* using a linear regression method is detailed in the Materials and Methods. In scatterplots in Figs 4 and 5, S is plotted along the x-axis. Along the y-axis S is multiplied by the log fold change in bulk RNAseq data after *tbx16* or *msgn1* overexpression (Figs 4 and 5 and S7 and S8 Tables). These plots provide a measure of how strongly perturbation-induced shifts in gene expression affect the random forest model-inferred probability of cell state transitions.

This analysis indicates that *tbx16* principally represses genes important for defining each transcriptional state. *Tbx16* represses genes that are normally repressed at the MP to PZ, PZ to pPSM and pPSM to aPSM transitions (upper left quadrants in Fig 4D, 4E and 4F). Moreover, *tbx16* most strongly represses genes that are the most important for defining each cell state. *Tbx16* represses both proneural genes that are normally upregulated at the NMP to neuronal transition and promesodermal genes are are downreguated at this transition (Fig 4B). *Tbx16* also represses genes that are normally upregulated and downregulated at the NMP to MP transition. *Tbx16* is known to repress *sox2*, which promotes NMP differentiation into neuronal precursors [28], but these data suggest that *tbx16* similarly promotes mesodermal progenitor differentiation into PZ and PSM via transcriptional repression.

*Msgn1* likewise represses gene expression but it affects far fewer genes (Fig 5). At the MP to PZ transition, *msgn1* almost exclusively represses genes that are normally downregulated at this transition. *Msgn1* also represses both proneural genes that are normally upregulated at the NMP to neuronal transition and promesodermal genes that are normally downregulated at this transition. Similarly, m*sgn1* represses both pro-NMP genes at the NMP to MP transitions and represses pro-MP genes (Fig 5B and 5C). These results again suggest that *msgn1* promotes paraxial mesoderm differentiation by repressing genes that are important for defining the MP cell state.

### The machine learning gene lists are enriched for important genes

We compared both the DESeq2 and machine learning gene lists for each cell state in wild-type tailbuds to the lists of DEGs in tg(*hsp70l:tbx16*) embryos and tg(*hsp70l:msgn1*) embryos. Since *tbx16* and *msgn1* are required for cells to mature into PSM, we focused on the MP to PZ transition and PZ to pPSM transition. Genes that are downregulated at these transitions in wild-type embryos and also repressed by overexpression of *tbx16* and *msgn1* were the most conspicuous in the scatterplots (Figs 4D, 4E, 5D and 5E), so we specifically list these genes in Table 1.

**Table 1:** Commonly and uniquely repressed genes by *tbx16* and *msgn1* overexpression at the MP to PZ and PZ to pPSM transition. All genes have been filtered by a 1.5-fold change threshold of expression level change. Genes or pathways required for body elongation and/or mesoderm differentiation (bold) and somitogenesis (underlined) are indicated.

Genes repressed during the MP to PZ transition													
Genes repressed by both *tbx16* and *msgn1*			Genes repressed by *tbx16* only								Genes repressed by *msgn1* only		
Machine learning and DESeq2	Machine learning only	DESeq2 only	Machine learning and DESeq2	Machine learning only	DESeq2 only						Machine learning and DESeq2	Machine learning only	DESeq2 only
adam8a **apela** **bmp2a** **fgf3** foxd3 **hoxa13b** rasl11b **tbxta** **tll1**	**hoxd12a** si:dkey-18j18.3	cdh6 esrrga **fgf4** hhla2a.1 hpcal4 itgb4 **lfng** s1pr1 si:dkey-40c11.2 usp54a **wnt2bb** zgc:110158	ankrd50l **eve1** **fgf8a** msx1b sox2 **sp9** tuba1c **wnt11**	enc3 hnrnpabb npm1a pcna rrm2 rtca tagln3b thbs2a timm50 tuba1c ube2e2 zgc:110158	b3gnt7l calm1b foxb1a griffin lrrc17 lypd6b mcm3 notch3 oflml2bb pax3a prr18 prrx1b				rcan1a rdh8a sox21a **wnt8a** wu:fc23c09		ubl3a	**cyp26a1** thbs2a	CABZ0176996.1 ephb4b marveld1 pou5f3 zgc:92140
**Genes repressed during the PZ to pPSM transition**													
**Genes repressed by both *tbx16* and *msgn1***			**Genes repressed by *tbx16* only**								**Genes repressed by *msgn1* only**		
**Machine learning and DESeq2**	**Machine learning only**	**DESeq2 only**	**Machine learning and DESeq2**	**Machine learning only**	**DESeq2 only**						**Machine learning and DESeq2**	**Machine learning only**	**DESeq2 only**
**hoxd12a** **tbxta**	inka1b	**bmp2a** **fgf24** foxd3 gdf11 **hoxa13b** kif26ab pip5k1ca si:dkey-18j18.3 sox11a thbs2a **tll1**	**bambia** **cdx4** hes6 itm2cb **msgn1** ptgdsb.1 rbm38 sst6 **tbx16l** **ved** **vent** **vox**	cx43.4s	adamts18 arhgap35b b3gnt7l blf ca8 **cdx1a** clip2 clstn1 col11a1a dhrs3b enc1 enc3 **eve1** **fgf10**	flrt3 **gdf3** her12 **hoxc11a** **hoxc11b** hoxd3a hoxd9a htr5aa inka1b lin28a macf1a mapkapk3 mcamb msx1a		mycn nid1b nt5dc2 parvb pde7a phkg2 pkl3 prkar2aa ptgdsb.2 ptk7a rarga rargb rdh8a shroom4		si:ch211-22614.6 si:dkey-261h17.1 si:dkey-44g23.5 **sp5l** **szl** tagln3b timm50 tob1a tubb5 **vimr1** **wnt11** ube2e2	**cyp26a1**		aebp1 **bmp4** CABZ01076996.1 cav1 **lef1** msx2b **sp5a** stxbp5a ubl3a
**Bold:** Genes or pathways required for body elongation.							Underlined: Genes or pathways required for somitogenesis						

**Fig 6:**
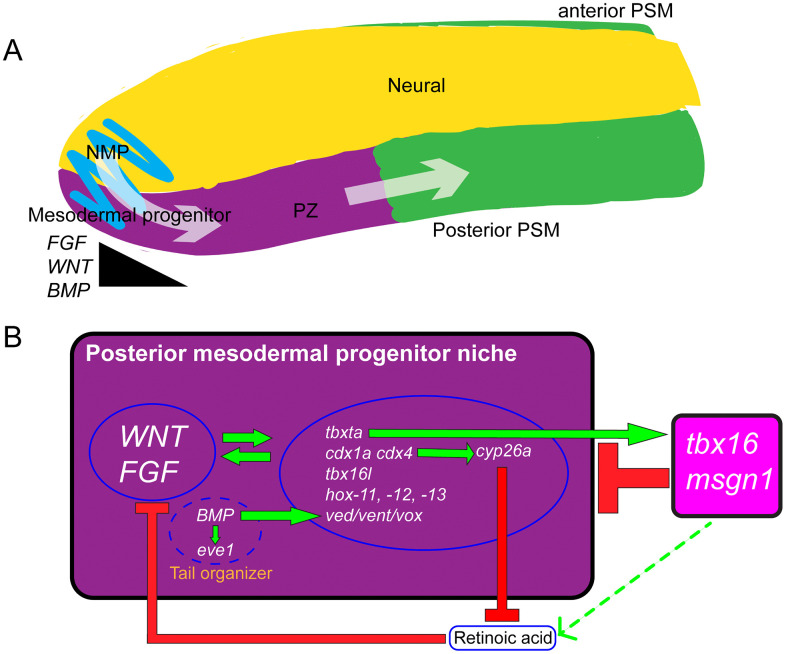
*Tbx16* and *msgn1* promote mesodermal differentiation by inhibiting the maintenance of the mesodermal progenitor cell state. **(A)** A schematic of cell state transitions in the zebrafish tailbud, from NMP to mesodermal progenitor to posterior PSM to anterior PSM. Gradients of FGF, Wnt and BMP signaling in the posterior tailbud promote body elongation, mesodermal fates and maintain the progenitor state. **(B)** The paraxial mesoderm gene regulatory network in which *tbx16* and *msgn1* repress expression of genes required to maintain the progenitor niche. This repression drives cell state transitions from mesodermal progenitors to PZ and PZ to posterior PSM. During body elongation, cells that are displaced away from the posterior tailbud escape the FGF, Wnt and BMP gradients enabling the negative feedback by *tbx16* and *msgn1* to predominate and commit the cells to differentiation.

We compared genes repressed by *tbx16* with *msgn1* in the MP to PZ and PZ to pPSM transitions, and we found both common and uniquely repressed genes (Table 1). Generally, *tbx16* has more unique downstream genes than *msgn1*. 74.19% of *msgn1* regulated genes are shared with *tbx16* at the MP to PZ transition, whereas only 38.33% of *tbx16* downstream genes are shared with *msgn1*. At the PZ to pPSM transition, 58.33% of *msgn1* downstream genes are shared with *tbx16*, whereas only 17.28% of *tbx16* regulated genes are shared with *msgn1*. Many of the repressed genes have known roles in body elongation, mesoderm differentiation and somitogenesis [8,9,16,27–29,41,54–70]. During the MP to PZ transition, *apela*, *bmp2a*, *fgf3*, *fgf4*, *hoxa13b*, *hoxd12a*, *lfng*, *tbxta, tll1,* and *wnt2bb* signaling are repressed by both *tbx16* and *msgn1*. *Tbx16* uniquely represses *eve1*, *fgf8a, sp9, wnt11* and *wnt8a*. *Msgn1* uniquely represses *cyp26a1* and *ephb4b*. During the PZ to pPSM transition, *bmp2a*, *fgf24*, *hoxa13b*, *hoxd12a, lef1,* and *tbxta* are repressed by both *tbx16* and *msgn1*. *Tbx16* uniquely represses *bambia*, *cdx1a*, *cdx4*, *eve1*, *fgf10a, gdf3*, *hes6*, *her12*, *hoxc11a*, *hoxc11b*, *hoxd3a, msgn1, sp5l, szl*, *tbx16l*, *wnt11, ved, vent, vimr1* and *vox*. *Msgn1* uniquely represses *bmp4, cyp261a* and *sp5a*. This analysis indicates that *tbx16* and *msgn1* repress transcription of genes that promote the mesodermal progenitor cell state (Fig 6).

## Discussion

### The Random Forest Classifier and SHAPLEY aid gene expression analysis

Here, we combined analysis of RNAseq and scRNAseq data to take advantage of the read depth of the former and the single cell resolution of the latter. However, a challenge with genomics experiments is extracting useful information from the resulting gene lists. We previously utilized UMAP to create a one-dimensional pseudotime for differentiation of paraxial mesoderm in the zebrafish tailbud. We then segmented the pseudotime into discrete transcriptional cell states using a variance minimization algorithm. Changes in these cell states after perturbation of BMP, Wnt or Fgf signaling can be quantitatively mapped onto the tailbud using multicolor fluorescent in situ hybridization [14]. Here, we used this pseudotime to help analyze the initial burst of gene expression after temporally controlled overexpression of *tbx16* and *msgn1*.

First, we use machine learning and DESeq2 in parallel to identify genes with significant expression level changes during cell state transitions in wild-type paraxial mesoderm differentiation. The two methods cross-validate each other because there is substantial number of genes that are identified by both methods (Table 1). Both methods support the conclusion that *tbx16* and *msgn1* promote mesodermal differentiation by inhibiting the maintenance of the mesodermal progenitor niche, though this conclusion is most evident in the machine learning analysis. Comparing the two methods with each other, we found that the machine learning method produced a shorter gene list. A premise of the machine learning analysis is that genes that display clear transitions in expression levels during paraxial mesoderm differentiation are particularly important for differentiation. This premise is validated by the observation that the machine learning approach is more enriched for genes with known functions in body elongation and mesoderm differentiation (Table 1). The DESeq2 pseudobulking method produced a larger gene list and included additional genes or pathways known to be required for body elongation and mesoderm development. However, the frequency of these known genes is lower than in the machine learning list suggesting that the DESeq list is noisier. Conversely, important genes with noisy expression or low expression that are poorly sampled by scRNAseq may be missed by the machine learning approach. Thus, the parallel application of these two methods is a powerful approach in gene expression analysis to identify possible target genes. The machine learning method is more selective but perhaps has more false negatives, and the DESeq2 method is likely noisier but more inclusive.

### *tbx16* and *msgn1* repress the mesoderm progenitor cell state

Previous studies indicate that *tbx16* and *msgn1* have both independent and redundant functions in paraxial mesoderm development. Loss of either *tbx16* or *msgn1* function leads to a failure of PSM differentiation with loss of *tbx16* having a much stronger phenotype [15–19]. Phenotypic differences are similarly evident after overexpression of *tbx16* or *msgn1*: *tbx16* overexpression has more severe elongation defect than *msgn1* overexpression (Fig 1C-1D) [9,18,28,49]. However, the heat-shock-inducible *msgn1* transgene in this paper produces weaker phenotypes than transgenes in these prior studies.

Several prior analyses utilized heat-shock-inducible *tbx16* and *msgn1* zebrafish transgenes to study mesodermal differentiation and gene regulatory networks [9,28,49]. In these studies, transgenic embryos were heat-shocked at 10–12 somite stage, and they exhibited less severe phenotypes than the embryos heat-shocked at 2–3 somite stage in our study. We also observe that later-stage heat-shock produces qualitatively similar but quantitatively weaker phenotypes. We chose to focus on early-stage heat shock because the tailbud dissections would isolate more tissue for RNA sequencing. These prior studies showed that Wnt and Fgf signaling induce NMPs to undergo an epithelial to mesenchymal transition and activate *tbx16* and *msgn1* expression downstream of *tbxta* as cells commit to a mesodermal fate. Meanwhile, *tbx16* and *msgn1* turn off the NMP state by repressing *sox2* and *tbxta*, making the transition to mesodermal fate irreversible. Our data show that *tbx16* and *msgn1* also repress multiple Wnt and Fgf pathway genes at the later MP to PZ and PZ to posterior PSM transitions. Therefore, *tbx16* and *msgn1* function with Wnt and Fgf signaling in a negative feedback loop that broadly regulates paraxial mesoderm differentiation. A similar regulatory network is involved in mouse NMP differentiation to mesoderm and includes *msgn1* but not *tbx16* [44,59].

Our analysis suggests that *tbx16* and *msgn1* promote mesodermal differentiation by inhibiting the maintenance of the mesodermal progenitor cell state which is consistent with prior analysis that examined a smaller number of genes (Fig 6) [18]. Wnt and Fgf signaling are key pathways required for the maintenance of mesodermal progenitor niche keeping them in an unsegmented state [71–76]. There is extensive crosstalk between the Wnt and Fgf signaling in the zebrafish tailbud [50,77]. Wnt and Fgf signaling also induce the mesodermal fates by activating t-box genes (*tbx16*, *tbxta*, and *tbx6*), *cdx* genes, and *ved/vent/vox* genes [8,9,16,27,28,41,58,59]. *Cdx* genes further activate *cyp26a* to repress retinoic acid (RA) signaling, which represses Wnt and Fgf pathway signaling in a negative feedback loop [29,59,60]. Posterior *hox* genes (*hox-11,-12,-13*) are also expressed in mesodermal progenitor cells and required for mesoderm formation and posterior body elongation [29,60]. In addition, the BMP signaling pathway also activates *ved/vent/vox* genes. It is the key pathway in the tail organizer, which regulates posterior body elongation, and cooperates with gradients of Wnt and Fgf signaling to control gene expression and cell motion [14,55,58,78–82]. All these pathways and related genes that maintain the mesodermal progenitor niche are repressed by *tbx16* and *msgn1* (Table 1 and Fig 6). This repression would drive irreversible cell fate transition from mesodermal progenitor cells to presomitic mesoderm. *Tbx16* and *msgn1* repress expression of many of the same genes, and *tbx16* represses more unique genes than *msgn1*. Thus, our data reveals both redundancy and independence of *tbx16* and *msgn1* of gene regulation in mesodermal differentiation (Table 1).

*Tbx16* overexpression extends the *tbx6* expression domain anteriorly compared to wild type embryos but leads to a 3-fold decrease in overall *tbx6* mRNA level (Fig 1F and S1 Table). An anterior extension of *tbx6* expression was also observed in *ripply1*; *ripply2* double mutant zebrafish embryos [83]. However, *tbx16* overexpression increases *ripply2* expression at 1.82-fold change compared to wild type, with no significant expression level change to *ripply1* (S1 Table). In mouse, *ripply* genes are activated by *mesp2*, and *mesp* genes interact with *notch* via negative feedback to establish somite boundaries [83,84]. In zebrafish, however, *ripply* and *mesp* genes act independently to regulate somite boundary formation [83]. *Tbx16* overexpression decreases the expression of *mespaa* (*mesp1*), *notch1b* and *notch 3* at 3 ~ 4-fold change but increases *mespba* expression at more than 2-fold change compared to wild type. Thus, it is currently unclear how *tbx16* overexpression directs the anterior extension *tbx6* expression domain.

### Differentiation by transcriptional repression

For both pluripotent stem cells and progenitor cells, there are several examples of differentiation by repressing genes that maintain a progenitor state. Embryonic stem cell differentiation to neural progenitor cells needs *polycomb*-mediated repressive epigenetic modification H3K27Me3 to silence *Jarid2*-sensitive genes [85]. Cortical neural stem cells require *sox10* to differentiate into oligodendrocyte precursors because *sox10* represses stem-cell programming factors such as *sox2* and *sox9* [86]. *Sox11*, expressed by oligodendrocytes progenitor cells, is epigenetically inhibited by Oligodendrocyte Transcription Factor 2 when the oligodendrocytes progenitor cells differentiate to immature oligodendrocytes [87]. Retinal progenitor cells require *Jarid2* to repress the early retinal cell gene *foxp1* to differentiate into late retinal progenitor cells [88]. Differentiation of epidermal progenitor cells necessitates a decrease in the expression of *SNAI2*, which represses differentiation and cell adhesion genes. If *SNAI2* expression is maintained, the epidermal progenitor cells will maintain the progenitor state [89]. Compared to these examples, *tbx16* and *msgn1* repress a larger number of genes within the mesodermal progenitor niche gene regulatory network. Therefore, the role of repression is broader, regulating the general process of posterior body elongation and formation of the trunk and tail mesoderm.

## Conclusion

In this study, we employ machine learning and game theory to analyze the gene network that governs zebrafish body elongation and paraxial mesoderm development. This analysis reveals that *tbx16* and *msgn1* promote mesodermal differentiation primarily by inhibition of the mesodermal progenitor gene regulatory network. This study contributes new insights not only into vertebrate body elongation and mesodermal development but also into the methodology of gene expression data analysis.

## Materials and methods

### Transgenic fish line preparation and heat-shock operation

#### Ethics Statement.

Tüpfel long fin zebrafish (wild-type fish) were raised according to standard protocols and experiments approved by the Institutional Animal Care and Use Committee. Transgenic lines were created using Tol2 transposase system [48]. For the tg*(pT2_Tol2_hsp70_tbx16_OPT_yCry_GFP_Tol2)* and *tg(pT2_Tol2_hsp70_msgn1_OPT_yCry_GFP_Tol2)* constructs (abbreviated as tg(*hsp70l:tbx16*) and tg(*hsp70l:msgn1*), the full length coding sequence of *tbx16* and *msgn1* was cloned with primers listed in S9 Table. Transgenic embryos were heat-shocked at 38°C for 30 minutes at the 2–3 somite-stage and raised for 24 hours post heat shock at 28.6°C to observe phenotypes. After robustness and consistency of phenotypes were confirmed in F1 transgenics, transgenic fish were in-crossed to produce homozygotes. F2 transgenics were out-crossed with wild type to screen by heat-shock for homozygotes and heterozygotes. For experiments, we outcrossed to wild type to either homozygotes for tg(*hsp70l:tbx16*) or heterozygotes for tg(*hsp70l:msgn1*).

### Quantitative PCR

qPCR experiments were performed for homozygous tg(*hsp70l:tbx16*) embryos 2, 3, 4, 5 hours post heat shock and wild-type embryos 2 hours post heat shock. Rabbit *beta globin* sequence in our transgene was used to measure heat shock induction of transgene expression. *Her1*, *her7*, *tbx6* were assayed as markers of paraxial mesoderm development and *beta-actin* was used as a control [50]. Primers are listed in S9 Table. 6 biological replicates, each with 3 technical replicates, were performed for each experimental condition. For each biological replicate, 10 embryos were pooled to extract RNA by QIAGEN RNeasy Plus Micro kit. To compare relative expression of the tg(*hsp70l:tbx16*) and tg(*hsp70l:msgn1*) transgenes 3 hours after heat shock, transgenic heterozygotes were crossed to TLF, heat shocked at the 2–4 somites stage and 40 embryos were pooled for RNA extraction for each of three biological replicates. A second set of β-globin primers were used for qPCR.

### *In Situ* hybridization

Probes, embryo processing and imaging parameters are described in [14]. Images were taken by the Leica Stellaris Falcon STED confocal microscope using a 20x objective.

### Tailbud dissection and RNA sequencing

Heat-shocked embryos were incubated for 3 hours after heat shock at 28.6°C and then dissected in ice-cold Hank’s balanced salt solution (HBSS). The tailbud was collected by cutting immediately posterior to the last formed somite. 10 tailbuds were collected per sample and 3 samples were prepared for per genetic background (tg(*hsp70l:tbx16*), tg(*hsp70l:msgn1*) and wild type). Since we used heterozygous tg(*hsp70l:msgn1*) fish, the anterior body of each dissected embryo was PCR genotyped using the rabbit *beta globin* UTR primers (S9 Table). Transgenic tailbuds were then pooled before RNA purification while non-transgenics tailbuds were pooled for wild-type heat shock controls. RNA was extracted for each sample with Trizol. Samples were sent to Yale Center for Genomic Analysis (YCGA) for RNA sequencing and data analysis. Low quality reads were trimmed, and adaptor contamination was removed using Trim Galore (v0.5.0). Trimmed reads were mapped to the zebrafish reference genome (GRCz11) using HISAT2 (v2.1.0) [90]. Gene expression levels were quantified using StringTie (v1.3.3b) [91]. Differentially expressed genes (DEGs) were identified using DESeq2 with a 0.05 false discovery rate (v 1.22.1) [92] This list was additionally filtered for genes with at least a 1.5 fold change in expression.

### DESeq2 analysis of expression changes

To identify genes differentially expressed over developmental pseudotime, we analyzed the scRNAseq data from [14]. Using Seurat v5 we pseudobulked by pseudotime derived cell-type and experimental replicate. We filtered the gene list to those expressed in at least 1% of cells in at least one cell-type giving a list of 10269 genes and then performed DESeq2 pairwise on each adjacent pseudotime segment. Genes with significant changes in expression at a transition were taken as those with at least a 1.5 fold change and an adjusted p-value of less than 0.05.

### Data-driven analysis of gene markers

A data-driven approach was developed to correlate the change in bulk gene expression with the developmental gene expression state transitions. This analysis follows a two-step framework. In the first step, single-cell RNA sequencing (scRNA-seq) data from wild-type embryos were analyzed to identify key genes that are strongly correlated with cell state transitions using a Random Forrest (RF) decision tree–based method. The contribution of each gene to single-cell state transitions was then quantified using Shapley values. The second step utilizes these Shapley values across all genes and cells to quantify how changes in bulk gene expression influence the prediction of cell gene expression states.

A flowchart summarizing the first step is shown in Fig 2D. The analysis utilizes scRNA-seq data from wild-type embryos. Gene expression state data from [14], identified by segmentation of pseudotime using a variance minimization algorithm, were used as the target labels. To identify informative genes, an initial Random Forest (RF) classifier was trained on the full gene-expression matrix, and genes were initially ranked according to their impurity-based feature-importance scores [52], also known as mean decrease impurity feature importance which reflect each gene’s cumulative contribution to classification across the RF ensemble. Based on this ranking, the top n genes were selected to construct reduced-feature RF models. Because impurity-based importance can be biased when features are highly correlated in gene-expression data [93], we further examined the contribution of each selected gene by calculating its Shapley value. The dataset was randomly divided into 80% training cells and 20% testing cells.

Starting with the single highest-ranked gene (n=1), the RF classifier was iteratively retrained while incrementally adding genes in descending order of impurity-based importance. For each iteration, five-fold cross-validation was performed on the training data, using four folds for training and one for validation. Predictive performance was quantified as the mean of the Area Under the receiver operating characteristic Curve (AUC) across all folds. The point of AUC convergence was used to determine the optimal number of genes required for accurate classification while mitigating sample-size imbalance effects.

During training, the RF hyperparameters, such as the number of estimators, maximum tree depth, minimum samples required for split, minimum samples per leaf, and the number of features considered at each split were jointly optimized with respect to the number of selected genes to maximize the AUC performance. In this work, when adding additional genes no longer improved the AUC, the corresponding subset of genes was selected as the final minimal gene set sufficient to define the respective cell state transitions. The resulting AUC convergence curves on the testing dataset and the corresponding optimal feature set sizes for each transition are presented in Fig 2F.

The final gene subsets identified by the decision tree-based approach contained 230,232,110, 55, and 235 genes for the transitions from NMP to neuronal, NMP to MP, MP to PZ, PZ to pPSM and pPSM to aPSM, respectively. Using the trained RF classifier structures and these optimized gene sets, we then applied the SHAP (Shapley Additive exPlanations) framework to compute Shapley values for each gene across all single cells in the dataset. The scatter plots in Fig 3B–F illustrate the 10 most influential genes for wild-type cell state transitions, ranked by their mean absolute Shapley values (left). The summary plots (right) integrate both feature importance and the directionality of each gene’s contribution to the cell state transition.

An essential component in the analysis of bulk gene expression is how the specific changes induced by the *tbx16* or *msgn1* compared to wild type could influence the transition of gene expression states. In the second step, an algorithm is developed to quantify the feature attribution when gene expression levels are scaled up or down by a constant factor k. Importantly, this algorithm facilitates comparisons between a gene’s specific fold change and influence on the gene expression states transition. Using the Shapley matrix calculated in the first step, we developed a log-scale sensitivity measure to quantify how changes in bulk gene expression affect the Shapley values in the cell state transitions.

Here, to normalize variation and reduce skewness of the expression value $x_{j}$ of a given gene in cell j(j=1,…,N), we log-transformed and standardized it into standardized expression $z_{j}$ by


$$\begin{array}{c} v_{j}={\text{log}}_{\text{2}}(x_{j}+\text{1}) \end{array}$$

$$\begin{array}{c} \mu_{v}=\frac{\text{1}}{N}\sum_{j=\text{1}}^{N}v_{j},\sigma_{v}=\sqrt{\frac{\text{1}}{N-\text{1}}\sum_{j=\text{1}}^{N}(v_{j}-\mu_{v}{)}^{\text{2}}} \end{array}$$

$$\begin{array}{c} z_{j}=\frac{v_{j}-\mu_{v}}{\sigma_{v}} \end{array}$$

where $v_{j}$ is the log-transform of the $x_{j}$, $\mu_{v}$ and $\sigma_{v}$ are the mean and variance of $v_{j}$. $x_{j}$ is added by 1 in the log2-fold change to avoid negative infinity for $v_{j}$.

We approximate the relationship between $z_{j}\;$and the corresponding Shapley value $\phi_{j}$using a local linear model:


$$\begin{array}{c} \phi (z)\approx \alpha +\beta z \end{array}$$

where the slope of this linear regression β is calculated by


$$\begin{array}{c} \beta =\frac{\text{Cov}(z,\phi )}{\text{Var}(z)} \end{array}$$

The expected change in the mean Shapley value resulting from scaling the group’s average expression $\bar{x}\;$by a factor k is then approximated by:


$$\begin{array}{c} \Delta (k)\approx \phi \left(\frac{{\text{log}}_{\text{2}}\left(k\bar{x}+\text{1}\right)-\mu_{v}}{\sigma_{v}}\right)-\phi \left(\frac{{\text{log}}_{\text{2}}\left(\bar{x}+\text{1}\right)-\mu_{v}}{\sigma_{v}}\right)\approx (\frac{\beta }{\sigma_{v}}){\text{log}}_{\text{2}}k \end{array}$$

Here, we define the log-scale sensitivity S  as:


$$\begin{array}{c} S=\frac{\beta }{\sigma_{v}} \end{array}$$

This term captures the proportional change in the Shapley value per log2-fold change in expression. Under this formulation, the relationship is symmetric, which indicates that increasing or decreasing expression by the same fold produces Shapley changes of equal magnitude but opposite direction:


$$\begin{array}{c} \Delta (\text{1}/k)=-\Delta (k) \end{array}$$

This log-scale sensitivity S  provides an interpretable, model-agnostic metric that enables comparability across genes with computational efficiency. Shapley sensitivity can be directly compared across different genes or cell states. The equation above offers a closed-form, linearized estimate of Shapley change without repeated model evaluation. This algorithm provides a fast and interpretable method for quantifying the responsiveness of model attributions to gene expression scaling in normalized log space.

In addition to the machine learning analysis of the bulk genes’ expression, the direct comparison of the bulk genes’ log fold changes by pseudobulk versus the genes’ expression changed in the wild-type single cell differentiation transitions stage is also compared in S2-S3 Figs.

### Identifying genes downstream of *tbx16* and *msgn1*

The genes list for each cell state transition in wild-type tailbuds were compared to the lists of DEGs in tg(*hsp70l:tbx16*) embryos and tg(*hsp70l:msgn1*) embryos, respectively, and common genes were found in each category. 1.5-fold-change threshold to all the gene lists was used. The common genes were then sorted by positive and negative fold change in each list. The number of genes in each category were counted, and all the gene names were listed.

## Supporting information

S1 Fig Overview of RNA sequencing of pooled dissected tailbuds from *tg(hsp70l:tbx16)*, and *tg(hsp70l:msgn1)* embryos. (A-B) Heatmap and MA plot of RNA-seq experiments comparing *tbx16* overexpression vs wild type. (C-D) Heatmap and MA plot of RNA-seq experiments comparing *msgn1* overexpression vs wild type. Each genotype has 3 biological replicates. All the identified differentially expressed genes (DEGs) have adjusted p-values < 0.05. Heatmaps are ranked by z-scores.
(TIF)

S2 FigTranscriptional regulation by *tbx16* during cell state transitions of paraxial mesoderm differentiation.(A) A schematic showing the developmental trajectory of the paraxial mesoderm in the tailbud. Body axes are shown as D (dorsal), V (ventral), A (anterior), P (posterior), R (right) and L (left). Cell state transitions are shown as NMP to neural, NMP to MP, MP to PZ, PZ to pPSM, and pPSM to aPSM. Each transition is labeled with an arrow indicating the direction of transition. (B-F) The logfold change of bulk gene expression induced by *tbx16* overexpression over wild type (y-axis) versus the logfold change of single-cell gene expression for the transitions in wild-type gene expression states (x-axis). Key genes identified by DESeq2 plus a 1.5-fold expression change threshold, either up- or downregulated (${\text{WT}}^{+},{\text{WT}}^{-}$), and overlapping with genes up- or downregulated (≥1.5-fold; $\text{tbx}{\text{16}}^{+},\text{tbx}{\text{16}}^{-}$) under *tbx16* overexpression are analyzed across the transitions from NMP to neural (B), NMP to MP (C), MP to PZ (D), PZ to pPSM (E), and pPSM to aPSM (F).(TIF)

S3 FigTranscriptional regulation by *msgn1* during cell state transitions of paraxial mesoderm differentiation.(A) A schematic showing the developmental trajectory of the paraxial mesoderm in the tailbud. Body axes are shown as D (dorsal), V (ventral), A (anterior), P (posterior), R (right) and L (left). Cell state transitions are shown as NMP to neural, NMP to MP, MP to PZ, PZ to pPSM, and pPSM to aPSM. Each transition is labeled with an arrow as the direction of transition (B-F) The logfold change of bulk gene expression induced by *msgn1* overexpression over wild type (y-axis) versus the logfold change of single-cell gene expression during wild-type cell state transitions (x-axis). Key genes identified by DESeq2 plus a 1.5-fold expression change threshold, either up- or downregulated (${\text{WT}}^{+},{\text{WT}}^{-}$), and overlapping with genes up- or downregulated (≥1.5-fold; ${\text{msgn1}}^{+},{\text{msgn1}}^{-}$) under *msgn1* overexpression are analyzed across the transitions from NMP to neural (B), NMP to MP (C), MP to PZ (D), PZ to pPSM (E), and pPSM to aPSM (F).(TIF)

S1 Table RNAseq results for tg(*hsp70l:tbx16*) compared to WT. (XLSX)

S2 Table RNAseq results for tg(*hsp70l:msgn1*) compared to WT. (XLSX)

S3 Table DESeq2 identification of differentially expressed genes at transcriptional cell state transitions. (XLSX)

S4 Table *Tbx16*-regulated genes identified with DESeq2 plotted in S2 Fig. (XLSX)

S5 Table *Msgn1*-regulated genes with DESeq2 plotted in S3 Fig. (XLSX)

S6 Table Machine learning identification and S values of differentially expressed genes at transcriptional cell state transitions. (XLSX)

S7 Table *Tbx16*-regulated genes identified with machine learning plotted in Fig 3. (XLSX)

S8 Table *Msgn1*-regulated genes identified with machine learning plotted in Fig 4. (XLSX)

S9 Table Primer Sequences. (XLSX)
